# Staying close to home: Ecological constraints on space use and range fidelity in a mountain ungulate

**DOI:** 10.1002/ece3.7893

**Published:** 2021-07-09

**Authors:** Yasaman N. Shakeri, Kevin S. White, Jason N. Waite

**Affiliations:** ^1^ Division of Wildlife Conservation Alaska Department of Fish and Game Juneau Alaska USA; ^2^ Wyoming Cooperative Fish and Wildlife Research Unit Department of Zoology and Physiology University of Wyoming Laramie Wyoming USA

**Keywords:** Alaska, GPS location data, home range, mountain goat, movement, *Oreamnos americanus*, range fidelity

## Abstract

Understanding patterns of animal space use and range fidelity has important implications for species and habitat conservation. For species that live in highly seasonal environments, such as mountain goats (*Oreamnos americanus*), spatial use patterns are expected to vary in relation to seasonal changes in environmental conditions and sex‐ or age‐specific selection pressures. To address hypotheses about sex, age, and seasonality influence on space‐use ecology, we collected GPS location data from 263 radio‐collared mountain goats (males, *n* = 140; females, *n* = 123) in coastal Alaska during 2005–2016. Location data were analyzed to derive seasonal and sex‐specific fixed‐kernel home range estimates and to quantify the degree of seasonal range and utilization distribution overlap. Overall, we determined that home range size was smallest during winter, expanded coincident with the onset of green‐up and parturition, and was largest during summer. Home range size of males and females did not differ significantly during winter, but females had larger home ranges than males during summer, a relationship that was switched during the mating season. Pairwise comparisons involving individual females across subsequent years indicated home ranges were significantly smaller during years when they gave birth to offspring. Mountain goats exhibited a strong degree of range fidelity, and 99% (*n* = 138) of individual animals returned to their previous year's seasonal range with an average annual Bhattacharyya's affinity utilization distribution overlap index of 68%. Similarity of seasonal home range utilization distributions varied in relation to sex and season in some respects. Home range overlap was highest during the summer vegetation growing season, particularly among females. These findings advance our understanding about how environmental variation and sex‐ and age‐related reproductive constraints influence space use and range fidelity among alpine ungulates. Documentation of the high degree of range fidelity among mountain goats has important conservation implications in landscapes increasingly altered by anthropogenic activities.

## Graphical abstract

We created seasonal and sex‐specific fixed‐kernel home range estimates to quantify seasonal home range size and utilization distribution overlap of mountain goats in southeast Alaska. We documented a high degree of range fidelity among mountain goats, which has conservation implications on a landscapes increasingly altered by anthropogenic activities.

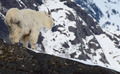

## 1 INTRODUCTION

Understanding how animals utilize critical habitat is important for advancing our understanding of species ecology and conservation. Technological innovations involving the advances in GPS transmitters, remote sensing, and computer processing have led to the development of powerful analytical approaches for delineating wildlife habitat at population‐level scales (Boyce et al., 2002; Cagnacci et al., 2010). However, examining how individual animals use given habitat patches provides an opportunity to attain a more complete understanding about habitat value and possible effects associated with anthropogenic alterations (Faille et al., 2010). For example, characterizing individual space‐use requirements and the extent to which such areas are consistently used can provide explicit knowledge about the value of a given habitat patch to an individual animal that can be linked to individual performance (Gerber et al., 2019; Lafontaine et al., 2017; Severud et al., 2015).

For species that live in highly seasonal environments, space‐use patterns are likely to vary due to changing environmental conditions as well as sex‐specific selection pressures (Bischof et al., 2012; Grignolio et al., 2004; Lesage et al., 2000; Lovari et al., 2006; Unterthiner et al., 2012; Yan et al., 2017; Zeng et al., 2010). Fundamental differences in fitness requirements between male and female individuals result in sex‐linked ecological variation, a pattern that is particularly evident among sexually dimorphic, polygynous mammals that display pronounced sex‐specific differences in nutritional ecology, social behavior, space use, and life‐history strategies (Barboza & Bowyer, 2000; Clutton‐Brock et al., 1982; Main et al., 1996). Such patterns arise because natural selection acts on males and females in separate ways due to fundamental differences in their reproductive characteristics (Darwin, 1888). Consequently, sex‐specific space‐use patterns are expected to vary seasonally, reflecting differences in reproductive requirements and associated trade‐offs related to energy balance and predation risk. For example, during the parturition season, smaller, more risk‐prone females with attendant neonates are expected to make space‐use decisions that prioritize predator avoidance, relative to larger‐bodied, less‐vulnerable males (Bonar et al., 2018; Miquelle et al., 1992; Van Beest et al., 2013).

The way in which space is used can have direct implications on fitness by influencing individual ability to acquire and conserve energetic resources and reduce predation risk (Severud et al., 2015). The amount of area used can be directly proportional to nutritional requirements (i.e., food biomass density) and costs (i.e., locomotory constraints), as well as availability of critical habitat features such as predator refugia—factors that can vary seasonally in relation to environmental conditions. The ability of animals to efficiently utilize resources or reduce predation risk within selected ranges may be optimized when individuals attain familiarity with such areas (Greenwood, 1980; Schaefer et al., 2000). Specifically, familiarity can play an important role in the habitat selection process at a broadscale and reinforce finer‐scale selection for beneficial attributes through positive reinforcement mechanisms (Wolf et al., 2009). As such, range fidelity, the seasonal or annual reuse of known ranges, has been widely documented in numerous species across three phyla (Switzer, 1993) and has been directly linked to measures of individual fitness. For example, range fidelity has been positively correlated with individual survival and reproduction in mammalian and avian species (Gerber et al., 2019; Lafontaine et al., 2017; Severud et al., 2015).

**FIGURE 1:**
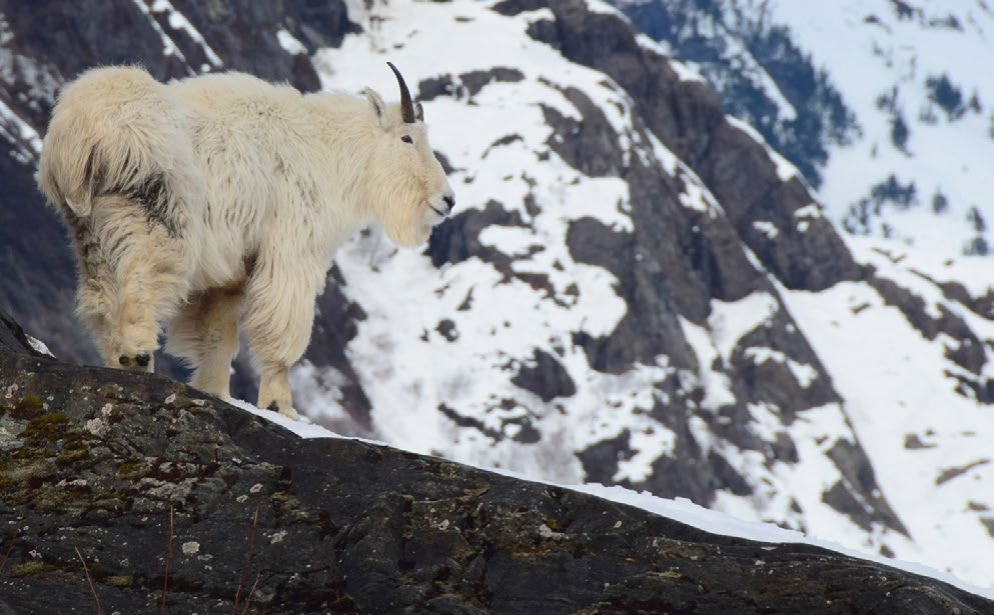
Mountain goat (*Oreamnos americanus*) in winter range. Southeast, Alaska

The study of species that inhabit extreme environments offers valuable opportunities to understand relationships between extrinsic and intrinsic factors influencing spatial use patterns and animal ecology. This occurs because selection pressure in such settings is often strong resulting in acute effects that often have important implications for conservation (Berger, 2018). In this regard, study of mountain ungulates, such as mountain goats (*Oreamnos americanus*), offers compelling case studies given their somewhat unique ecological attributes and specialized adaptations for surviving in rugged mountain environments characterized by extreme seasonal climates (Figure 1). The species exhibits a strong affinity for steep, broken terrain and cliffs in order to mitigate the risk of predation by large carnivores such as wolves, mountain lions, and bears (Cote et al., 1997; Festa‐Bianchet & Côté, 2008; Fox & Streveler, 1986; Smith, 1983). Such features are often limited in distribution and result in concentrated use of specific areas in mountain landscapes (Lowrey et al., 2018; Wells et al., 2014; White & Gregovich, 2018). Within this context, seasonal variability in food resource availability and energetic constraints imposes restrictions on how mountain goats use their environment. For example, the heavy, wet snow packs common in coastal environments increase the energetic costs of locomotion (Dailey & Hobbs, 1989), while decreasing the availability of forage (Fox, 1983; White et al., 2009) during winter. Such environmentally driven constraints can impose strong selection pressures illustrated by use of energy minimizing behavioral strategies during the winter and conservative life‐history characteristics (Festa‐Bianchet & Côté, 2008). The timing of reproductive effort differs between females and males, with females giving birth at the onset of the vegetation growing season and incurring associated energetic demands through mid‐summer (Hamel & Côté, 2009; Pettorelli et al., 2007). Polygynous males, on the other hand, widely seek and procure mating opportunities during the rutting season which occurs just prior to winter (Geist, 1965; Mainguy & Côté, 2008; Richard et al., 2014a). During the rut, males are expected to increase movement behavior and expand space use in order to optimize opportunities to find receptive females (Richard et al., 2014a).

The objective of this study was to examine how environmental seasonality, reproductive constraints, and behavioral factors influence space use and range fidelity of mountain goats, a habitat specialist that inhabits a highly seasonal mountain environment. Specifically, we predicted that space use would be highest during the vegetation growing season and lowest during snowy winters. Within this context, we expected sex‐specific differences such that females would exhibit restricted space use during the parturition period but would expand through summer as vulnerable neonates attained greater mobility, enabling females to more effectively acquire food resources. We predicted that space use would expand for males during the late‐fall breeding season in order to find and procure mating opportunities, a behavior that would be positively correlated with individual age. Because mountain goats are habitat specialists that utilize terrain features, or antipredator refugia, that are limited on the landscape, we expected individuals to exhibit a high degree of range fidelity. We expected higher range fidelity during critical life cycle periods (i.e., females during parturition and males during the breeding season), as compared to other times of year, patterns that would be generally expected to be more pronounced for older animals that had greater familiarity with their local environments.

## 2 STUDY AREA

**FIGURE 2:**
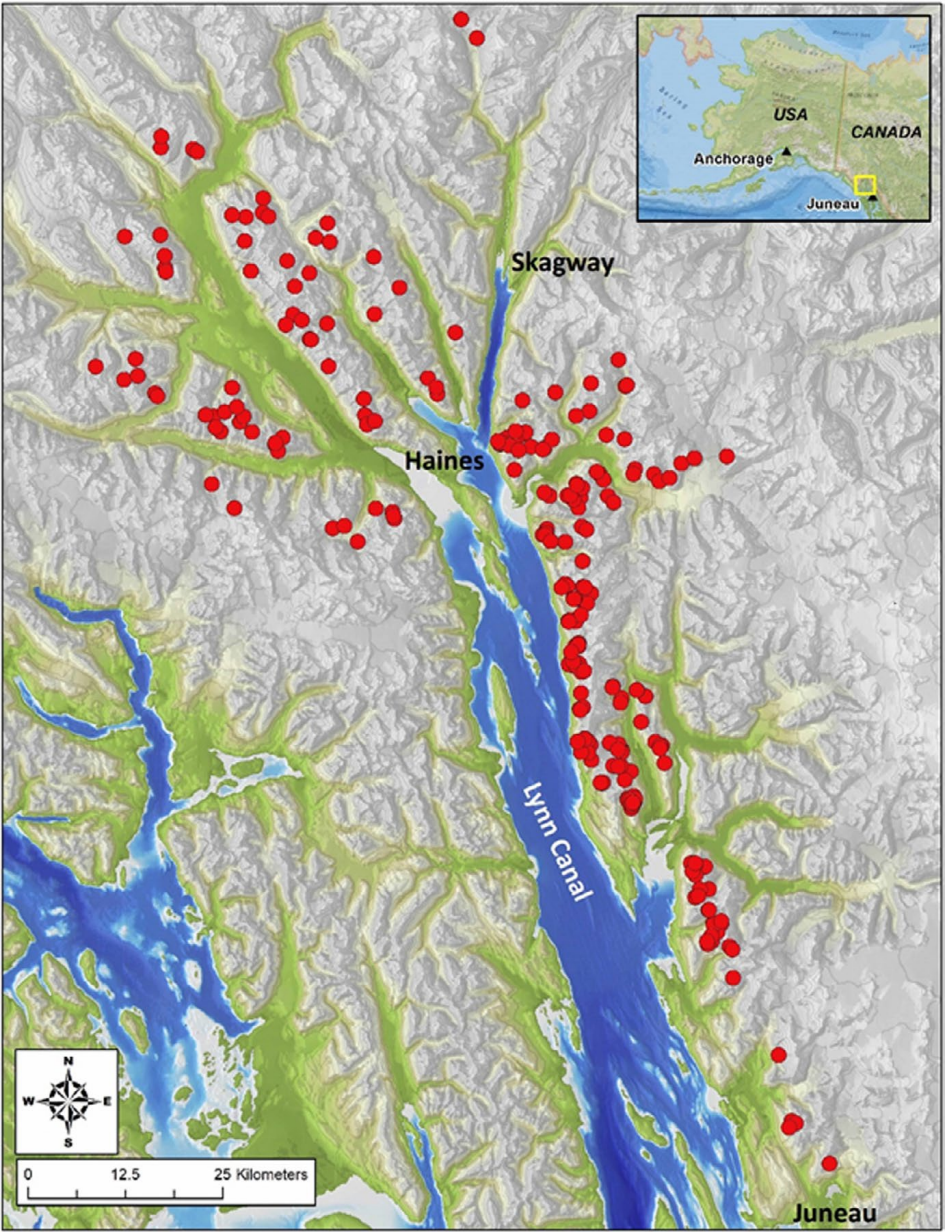
Map of the study area illustrating the location where mountain goats were studied during 2005–2016 in the upper Lynn Canal region, Alaska. Red circles denote mountain goat capture locations (*n* = 263)

We studied mountain goats in a 4,100‐km^2^ study area in a mainland coastal mountain range in the upper Lynn Canal region of southeastern Alaska, near the community of Haines, Alaska (58.4–59.7N, 134.8–136.2W; Figure 2). The maritime climate in this area is characterized by cool, wet summers and relatively warm, snowy winters. Total annual precipitation at sea level averages 159 cm, including 447 cm of snowfall deposited during November–March (Haines COOP Weather Station, AK; National Weather Service, 2020). Elevations at 800 m typically receive ca. 650 cm of snowfall, annually (Eaglecrest Ski Area Juneau, AK; National Weather Service, 2020). Winter temperatures at sea level average −3°C (and are rarely less than −20°C), whereas summer temperatures average 14°C (and rarely exceed 27°C; Haines COOP Weather Station, AK; National Weather Service, 2020). Predominant vegetative communities occurring at low‐to‐moderate elevations (<500 m) include Sitka spruce (*Picea sitchensis*)—western hemlock (*Tsuga heterophylla*) coniferous forest, mixed‐conifer muskeg, and deciduous riparian forests. Mountain hemlock (*Tsuga mertensiana*) dominated “krummholz” forest comprises a subalpine, timberline band occupying elevations between 500 and 750 m. Alpine plant communities (750–1,400 m) are composed of a mosaic of relatively dry ericaceous heathlands, moist meadows dominated by grasses and forbs, and wet fens. Avalanche chutes are common in the study area, bisect all plant community types, and often terminate at sea level. Mountain goats are largely allopatric with other potential interspecific competitors such as moose (*Alces alces*). Sitka black‐tailed (*Odocoileus hemionus sitchensis*) deer are rare. Documented predators of mountain goats include wolves (*Canis lupus*), brown bears (*Ursus arctos*), and black bears (*Ursus americanus*). Wolverines (*Gulo gulo*) and coyotes (*Canis latrans*) are also present.

## 3 METHODS

During August–October 2005–2016, mountain goats were chemically immobilized using standard helicopter darting techniques (Taylor, 2000; White et al., 2012). During handling, all animals were carefully examined and monitored following standard veterinary procedures (Taylor, 2000) and routine biological samples and morphological data collected. Age of animals was determined by counting horn annuli (Brandborg, 1955; Smith, 1988) and, in some cases, cross‐validated by examination of tooth eruption patterns (for young animals; Brandborg, 1955) and/or cementum analysis of incisors (for deceased animals; Matson, 1985). Individuals were subsequently classified into 3 a priori age classifications (subadult: 2–3 years, adult: 4–9 years, senior: 10+ years) based on age‐specific patterns of physical and sexual maturity and senescence (Festa‐Bianchet & Côté, 2008; White et al., 2011). All animals were deployed with GPS radio‐collars (TGW‐3590, TGW‐4500, or TGW‐4590, Telonics Inc., Mesa, AZ). Radio‐collars were programmed to collect GPS location data at 6‐hr intervals. Location data were postprocessed and filtered for “impossible” points and 2D locations with PDOP (position dilution of precision) values greater than 10, following (D'Eon & Delparte, 2005; D'Eon et al., 2002). During each location attempt, ancillary data about collar activity (i.e., percent of 1‐s switch transitions calculated over a 15‐min period following each GPS fix attempt) were simultaneously collected. Seasons were defined by using remotely collected activity sensor data as a proxy for defining behaviorally mediated changes in seasonal activity patterns, timing of migratory movements, or, in the case of kidding season, aerial observations of newborn neonates (winter: 15 December–15 April, kidding: 15 May–15 June, summer: 16 June–30 September, and rut: 18 October 18–23 November; White, 2006; White et al., 2012).

GPS location data were analyzed for each individual by season, provided at least 100 GPS locations were available. We estimated seasonal home range utilization distributions (95% fixed kernel; Worton, 1989) with the *href* (reference bandwidth) smoothing parameter using the rhr package (Signer & Balkenhol, 2015) in statistical program R (R Development Core Team, 2017). We used two complementary approaches to characterize fidelity to seasonal home ranges. First, we examined whether 95% fixed‐kernel seasonal home range polygons overlapped from one year to the next. Next, we quantified the degree to which spatial use patterns within home ranges (i.e., utilization distributions) were similar by comparing seasonal home ranges during successive years. Specifically, we used Bhattacharyya's affinity (BA) estimator, a computational method that quantifies similarity between paired utilization distributions (Bhattacharyya, 1943; Fieberg & Kochanny, 2005). Specifically, BA characterizes the utilization distribution overlap by deriving a continuous affinity measurement between 0 and 1 (i.e., 0 = no overlap, and 1 = complete overlap) (Calenge, 2011). BA estimates were derived by comparing a season‐specific utilization distribution for a given individual during year *t* + 1, with the corresponding season‐specific utilization distribution from the prior year (year *t*), thus providing an explicit assessment of the degree to which prior use of a given home range was emulated the following year. We used the kerneloverlaphr function available in the adehabitatHR package (Calenge, 2011) in program R to calculate estimates of BA.

### 3.1 Statistical analysis

A generalized linear mixed‐effect model was used to examine the effects of sex, age class, and season on home range size using the glmmPQL function in the MASS package (Venables & Ripley, 2002) in program R. Individual animal ID was included as random effect. We used a Gamma error distribution with a log link function to address both nonlinearity and heteroscedasticity in the response and Tukey‐adjusted post hoc pairwise comparisons to examine differences within groups. To determine whether kid status influenced home range size, we conducted a paired *t* test to contrast individual adult female goats during a year when they had a kid, with a year when they did not. The effect of sex, season, and age class on range fidelity (BA) was evaluated using a fractional response regression model implemented as a generalized linear model with a quasibinomial error distribution (Clark, 2019; Gourieroux et al., 1984; Papke & Wooldridge, 1996) using the mgcv package (Wood, 2017) in program R. Individual animal and year treated as random effects. We used Tukey‐adjusted post hoc pairwise comparisons to examine differences between all categorical combinations (season, age class, and sex). Categorical summaries were described by presenting mean ± *SE*.

## 4 RESULTS

During August–October 2005–2016, 263 mountain goats were captured and deployed with GPS radio‐collars and resulted in compilation of 171,685 GPS locations (GPS fix success = 83%). Seasonal home range estimates (95% fixed kernel) were calculated for 123 females and 140 males (mean number of locations/home range estimate = 241), in many cases across multiple years (mean deployment time = 1.7 years). Data were adequate to compare seasonal home range and utilization distribution overlap during consecutive years in a subset of cases (females, *n* = 67–118; males, *n* = 85–134, depending on season).

### 4.1 Home range size

**FIGURE 3:**
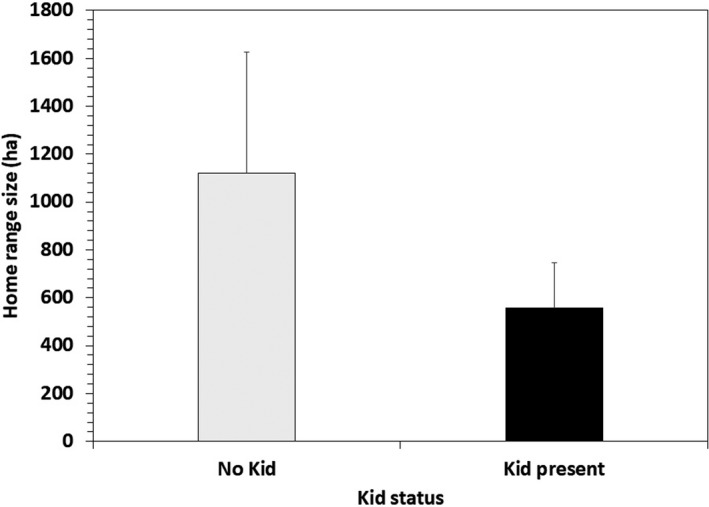
Home range size (95% fixed kernel; hectares) of adult female mountain goats during the kidding season in relation to kid status during 2005–2016 in Upper Lynn Canal, Alaska. Analyses include only paired cases involving individuals that had a kid one year and did not have a kid in the preceding or subsequent year (*p* = .05, *t* = 1.74, *n* = 14)

The model examining sources of variation in mountain goat home range size indicated that season, sex, and age class were all important predictors (Tables 1 and 2). Home range size was generally smallest during winter, as compared to other seasons (Table 2). Adult males tended to have the smallest winter home ranges (mean ± *SE* = 296 ± 28 ha, *n* = 152) and were 0.73 times smaller than adult females (mean ± *SE* = 407 ± 46 ha, *n* = 109, *p* = .03) and 0.57 times smaller than subadult male winter home ranges (mean ± *SE* = 519 ± 115 ha, *n* = 23, *p* = .04) than adult males. In contrast, during the kidding season, adult female home ranges (mean ± *SE* = 730 ± 92 ha, *n* = 81) were significantly (0.44 times) smaller than adult male home ranges (mean ± *SE* = 1,654 ± 176 ha, *n* = 116; *p* < .001). Further, analyses of paired comparisons of individual females indicated that adult females had significantly smaller home ranges (mean ± *SE* = 558 ± 189 ha, *n* = 14) during kidding seasons when they had an offspring at heel, as compared to kidding seasons when they did not have an offspring (mean ± *SE* = 1,121 ± 506 ha, *n* = 14; *t* = 1.74, *p* = .05; Figure 3). During summer, adult female home ranges expanded (mean ± *SE* = 1,303 ± 148 ha, *n* = 105) and were 1.5 times larger than adult male home ranges (mean ± *SE* = 875 ± 82 ha, *n* = 159, *p* = .007); subadult male home range size (mean ± *SE* = 1,906 ± 476 ha, *n* = 18) was significantly larger than adult male home range during summer (*p* = .007; Tables 1 and 2). During the breeding season, or rut, adult male home ranges (mean ± *SE* = 2,243 ± 213 ha, *n* = 151) were 2.5 times larger, as compared to summer (*p* < .001), and were significantly larger than adult females (mean ± *SE* = 606 ± 70 ha, *n* = 101; *p* = .008); age‐related differences were not evident within sex classes during this period (Tables 1 and 2).

**TABLE 1:** The parameter estimates, standard error, and statistical significance of the covariates used in the models for describing mountain goat (a) home range size and (b) home range fidelity (utilization distribution overlap; BA) in relation to sex, age class, and season during 2005–2016 in upper Lynn Canal, Alaska

Coefficients	(a) Home range size			(b) Home range fidelity		
	Estimate	*SE*	*p*‐value	Estimate	*SE*	*p*‐value
Intercept	6.594	0.125	<.001	0.869	0.159	<.001
senior	0.165	0.282	.559	−0.332	0.292	.255
subadult	0.329	0.369	.372	0.112	0.622	.858
rut	0.817	0.164	<.001	−0.356	0.188	.058
summer	−0.187	0.143	.190	0.899	0.205	<.001
winter	0.579	0.141	<.001	0.027	0.187	.884
male	−0.585	0.140	<.001	−0.074	0.200	.710
senior * rut	−0.529	0.400	.186	0.137	0.404	.736
subadult * rut	−0.628	0.558	.261	−0.461	0.700	.510
senior * summer	0.377	0.363	.299	−0.434	0.424	.307
subadult * summer	−0.300	0.409	.463	−0.248	0.781	.751
senior * winter	−0.022	0.357	.952	−0.215	0.378	.571
subadult * winter	−0.292	0.426	.493	−0.193	0.710	.786
senior * male	−0.032	0.352	.929	0.284	0.397	.474
subadult * male	−0.310	0.407	.446	−0.749	0.753	.320
rut * male	0.492	0.185	.008	0.332	0.248	.181
summer * male	−1.215	0.183	<.001	−0.417	0.263	.113
winter * male	−1.134	0.183	<.001	−0.667	0.245	.007
senior * rut * male	0.326	0.504	.518	0.067	0.538	.901
subadult * rut * male	0.939	0.616	.128	0.545	0.945	.565
senior * summer * male	0.535	0.495	.281	0.172	0.549	.755
subadult * summer * male	1.370	0.633	.031	−0.788	0.431	.141
senior * winter * male	0.873	0.499	.081	−0.328	0.513	.523
subadult * winter * male	1.168	0.611	.056	1.434	0.972	.140

Note

Intercept represents the baseline values (adult females during the kidding seasons). All other parameter estimates are relative to these baseline values.

**TABLE 2:** Mountain goat home range (95% fixed kernel) estimates in relation to sex, age category, and season during 2005–2016 in upper Lynn Canal, AK

Age	Sex	Season	Home range size (ha)					Group
			Mean	SE	Lower	Upper	*n*	
Subadult	Female	Kid	1,015	360	503	2,039	9	CDEFGHI
		Summer	1,352	380	781	2,345	15	BCDEF
		Rut	624	149	392	1,002	21	GHIJK
		Winter	415	96	262	659	22	JKL
	Male	Kid	1,227	505	545	2,752	6	BCDEFGH
		Summer	1,906	476	1,164	3,134	18	ABCD
		Rut	3,151	744	1,978	5,014	20	A
		Winter	519	115	334	804	23	IJK
Adult	Female	Kid	730	92	573	935	81	GHI
		Summer	1,303	148	1,043	1,636	105	CDE
		Rut	606	70	483	758	101	HIJ
		Winter	407	46	327	508	109	K
	Male	Kid	1,654	176	1,339	2,039	116	C
		Summer	875	82	728	1,054	159	FG
		Rut	2,243	213	1,863	2,697	151	AB
		Winter	296	28	245	358	152	L
Old	Female	Kid	861	226	513	1,451	16	EFGHI
		Summer	1,503	380	916	2,465	18	BCDE
		Rut	1,042	271	626	1,737	18	CDEFGH
		Winter	465	115	284	758	20	IJKL
	Male	Kid	1,149	310	672	1,959	15	CDEFG
		Summer	1,015	230	652	1,588	22	DEFG
		Rut	3,149	727	1,998	4,964	21	A
		Winter	478	113	299	758	20	IJKL

Note

All groups (sex, season, and age class) that share the same letter are not significantly different at alpha = 0.05 (Tukey‐adjusted post hoc pairwise comparisons).

### 4.2 Range fidelity

**FIGURE 4:**
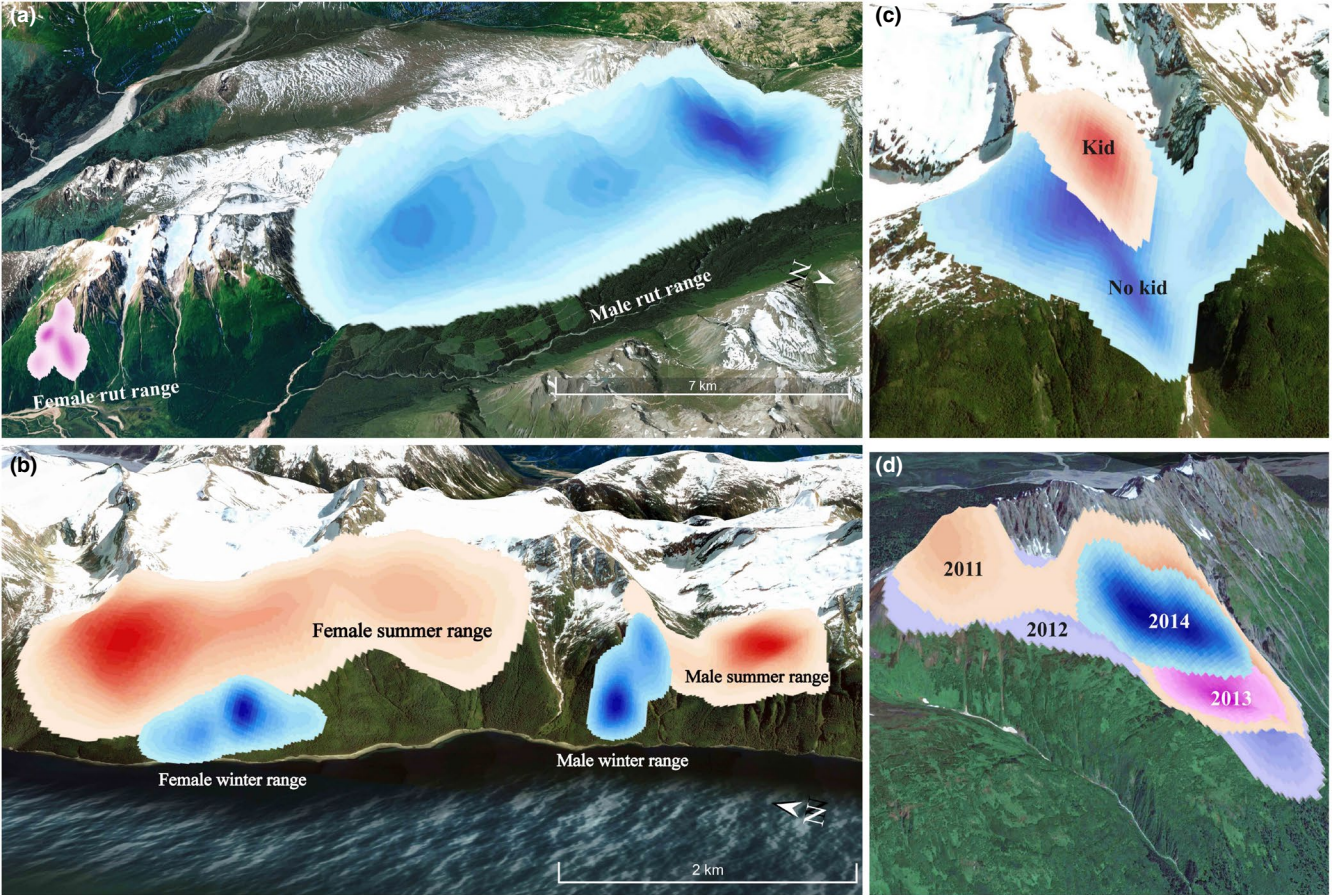
Maps depicting examples of sex‐ and season‐specific variation in mountain goat space use and site fidelity in upper Lynn Canal, Alaska during 2005–2016. (a) home range size of a representative male and female mountain goat during the rut, (b) differences in summer versus winter home ranges for a male and female mountain goat, (c) kidding season home range size of a female mountain goat during a year when it had a kid versus a year when it did not, (d) overlapping home ranges of a male mountain goat during 4 successive summer seasons illustrating site fidelity

Overall, seasonal home range overlap of mountain goat home ranges showed little variation during consecutive years. Specifically, 97% (810/836) of seasonal home ranges, at least partially, overlapped with the seasonal home range used during the previous season (Table 3). Yet, the specific manner in which utilization distributions were spatially emulated from one year to the next varied in relation to sex and season and, to a lesser extent, age. Specifically, the fractional response regression model indicated that season and sex were the most influential predictors of utilization distribution overlap (BA) or range fidelity (Tables 1 and 3 and Figure 4).

**TABLE 3:** Mountain goat home range and utilization distribution overlap (BA) estimates in relation to sex and season during 2005–2016 in upper Lynn Canal, Alaska

Age	Sex	Season	Home range overlap		Utilization distribution overlap					
			Cases	%	Mean	*SE*	Lower	Upper	*n*	Group
Subadult	Female	Kid	3	100	0.71	0.12	0.43	0.89	3	ABCDE
		Summer	7	100	0.83	0.07	0.66	0.93	7	BCDE
		Rut	9	100	0.54	0.08	0.39	0.69	9	ABCD
		Winter	9	100	0.70	0.07	0.54	0.82	9	ABCDE
	Male	Kid								
		Summer	3	100	0.60	0.13	0.34	0.82	3	ABCDE
		Rut	4	100	0.59	0.12	0.35	0.79	4	ABCDE
		Winter	4	100	0.70	0.11	0.46	0.86	4	ABCDE
Adult	Female	Kid	48	100	0.70	0.03	0.63	0.76	48	BC
		Summer	88	100	0.85	0.02	0.81	0.88	88	CD
		Rut	71	93	0.63	0.03	0.58	0.68	76	A
		Winter	89	100	0.71	0.02	0.67	0.76	89	BC
	Male	Kid	64	97	0.68	0.03	0.62	0.73	66	BCD
		Summer	106	100	0.78	0.02	0.74	0.81	106	BC
		Rut	87	100	0.68	0.02	0.63	0.73	87	AB
		Winter	72	86	0.54	0.03	0.49	0.59	84	DE
Old	Female	Kid	16	100	0.62	0.06	0.50	0.73	16	BCDE
		Summer	14	100	0.72	0.06	0.59	0.82	14	CDE
		Rut	13	93	0.56	0.07	0.43	0.69	14	ABCD
		Winter	20	100	0.57	0.06	0.46	0.67	20	CDE
	Male	Kid	19	100	0.68	0.05	0.57	0.77	19	BCD
		Summer	25	100	0.72	0.04	0.63	0.80	25	ABCD
		Rut	23	100	0.72	0.05	0.62	0.80	23	ABCD
		Winter	16	73	0.40	0.05	0.30	0.50	22	E

Note

Home range overlap summarizes whether a seasonal home range overlapped with the previous season. Utilization distribution overlap characterizes the similarity of seasonal home range utilization distributions for given individual during consecutive years, calculated using Bhattacharyya's affinity (BA) estimator. All groups (sex, season, and age class) that share the same letter are not significantly different at alpha = 0.05 (Tukey‐adjusted post hoc pairwise comparisons).

Utilization distribution overlap (BA) was highest during summer, as compared to other seasons, with adult females (BA, mean ± *SE* = 0.85 ± 0.02, *n* = 88) exhibiting a significantly greater degree of range fidelity than adult males (BA, mean ± *SE* = 0.78 ± 0.02, *n* = 106; *p* = .006); old females exhibited lower range fidelity than adult females (BA, mean ± *SE* = 0.72 ± 0.06, *n* = 14; *p* = .027) but age differences were not otherwise evident within sexes during summer (Table 3, Figure 4). A similar pattern of sex‐specific differences was evident during winter, with females (BA, mean ± *SE* = 0.71 ± 0.02, *n* = 89) exhibiting a higher degree of range fidelity than males (BA, mean ± *SE* = 0.54 ± 0.03, *n* = 84; *p* < .001), though in both cases utilization distribution overlap was lower in winter, as compared to summer (Table 3). Further, both old females (BA, mean ± *SE* = 0.57 ± 0.06, *n* = 20) and old males (BA, mean ± *SE* = 0.40 ± 0.05, *n* = 22) exhibited lower range fidelity in winter, as compared to adults in their respective sex class (females, *p* = .034; males, *p* = .041). Otherwise, males and females reused home ranges during the kidding and breeding season in a statistically indistinguishable manner (Table 3).

## 5 DISCUSSION

Mountain goats exhibited sex‐, age‐, and season‐specific variation in space use and range fidelity highlighting the role that environmental conditions, reproductive constraints, and associated social behavior play in influencing movement ecology in mountain‐adapted habitat specialists. In general, space use expanded during the vegetative growing season and constricted during the winter. Mountain goats inhabit winter environments characterized by relatively extreme snow conditions that constrain food resource availability and increase costs of locomotion. Consequently, mountain goats employ relatively extreme energy conservation strategies characterized by restricted movement and space use during winter (i.e., roughly 30% the size of summer home ranges), a finding consistent with previous studies of mountain goats (Keim, 2004; Poole et al., 2009; White, 2006) and other ecologically comparable mountain ungulate species (Grignolio et al., 2007; Poole et al., 2016; Simmons, 1982). Such results contrast with relationships documented in other large herbivore species, where winter home range constriction is more variable and less pronounced or absent (Mysterud et al., 2001). During the summer vegetative growing season, mobility and availability of food resources increase and mountain goats significantly expand space use, a strategy expected to promote accumulation of endogenous energetic reserves necessary for growth, reproduction, and overwinter survival (Parker et al., 2009). Overall, the expression of seasonal variability in space use in response to changing environmental conditions is common among northern ungulates (Van Beest et al., 2011), yet the relatively high degree of seasonal change in space use between summer and winter among mountain goats is notable and provides insight about how seasonality influences movement patterns among habitat specialists inhabiting mountain landscapes.

### 5.1 Sex‐ and season‐specific effects on home range size

Variation in sex‐specific reproductive characteristics influenced space‐use strategies among mountain goats. Home range sizes were small and did not differ among sexes during winter. However, similar to other northern ungulates in predator‐rich systems (Grignolio et al., 2004; Severud et al., 2015; Testa et al., 2000; Van Beest et al., 2011), female home ranges during the parturition season were smaller than males, particularly among those that had attendant neonates. In coastal Alaska, mountain goats typically migrate between low elevation winter ranges to higher‐elevation alpine summer habitats (White et al., 2012). For females, migration immediately precedes parturition, with females giving birth in spatially isolated (and putatively safer; i.e., Bergerud et al., 1984) alpine habitats between mid‐May to early‐June, often prior to alpine green‐up in snow‐covered settings. Males, lacking reproductive constraints of parturition, conduct slower and somewhat delayed altitudinal migrations, potentially to more closely track green waves (Bischof et al., 2012) of emergent vegetation. As a consequence, male home ranges during the kidding season are large because they occur during the spring migratory period and often encapsulate portions of winter and summer range. During the postkidding summer period, female home ranges expand and exceed the size of male home ranges. Following an initial period of neonate vulnerability and spatial constriction associated with birth events, expansion of female home ranges during summer coincides with the need to cope with increased energetic demands associated with lactation and recovery fat and protein reserves depleted over winter (Parker et al., 2009) and also reduce the risk of predation. Indeed, space‐use expansion among females during summer is expected to result in more dispersed and less predictable use of the landscape, characteristics that are expected to reduce the risk of predation among mountain goats (Festa‐Bianchet & Côté, 2008) and other large herbivores in circumboreal regions (Bergerud et al., 1984; Bowyer et al., 1999). Overall, these findings reinforce the important role that neonate vulnerability and energetic costs of lactation play in regulating seasonal variation in home range size.

The mating season imposes sex‐specific constraints on mountain goats and resulted in different space‐use strategies during the rut. Successful breeding among polygynous male mountain goats involves finding and breeding with receptive females during an abbreviated 4‐ to 6‐week period during late autumn to early winter. Reproductive success among males is linked to body mass and age class, with larger (and older) males typically being more socially dominant and successful breeders, as compared to younger animals (Mainguy & Côté, 2008). Our results indicated that males substantially expanded home ranges during the rut, consistent with the need to find multiple receptive females across relatively broad mountain landscapes where densities can be variable and patchily distributed. Females, on the other hand, reduced home ranges size during the rut, as compared to summer. Such behavior may facilitate the ability of males to find receptive mates during the rutting season by concentrating use in relatively restricted areas, as has been documented in white‐tailed deer (Beier & McCullough, 1990).

### 5.2 Seasonal patterns of home range fidelity

Range fidelity has been proposed to confer ecological benefits because increased familiarity with an area is predicted to enhance acquisition of nutritional resources and reduce predation risk (Greenwood, 1980; Schaefer et al., 2000), though predictable prey behavior may increase predation risk in some settings (Bowyer et al., 1999). Quantitative assessment of range fidelity by statistically comparing similarity of utilization distributions, using methods such as Bhattacharyya's affinity (BA) estimator, offers an informative means for characterizing the ecological underpinnings of range fidelity and has been used in a diversity of systems (Caillaud et al., 2014; Clapp & Beck, 2015; Hartman et al., 2015; Kochanny et al., 2009; Naveda‐Rodríguez et al., 2018; Robert et al., 2012; Sansom et al., 2018; Watson et al., 2014). Our findings indicate that at a broadscale mountain goats exhibit a high degree of range fidelity such that in nearly all cases (i.e., 99%, *n* = 138) individuals had overlapping seasonal home ranges from one year to the next, a finding comparable to earlier, less detailed study of the species (Keim, 2004; Schoen & Kirchoff, 1982; Swenson, 1985). The average annual BA overlap for all individuals during all seasons was 68% (*n* = 138). This finding is notable considering that migratory mountain goats in the Lynn Canal region of our study area exhibited annually recurrent use of largely the same 3–4 km^2^ winter home ranges despite having as much as 106 km^2^ of winter range available (White, 2006; White et al., 2012; White and Gregovich, 2017; Figure S1). Within this context, finer resolution utilization distribution similarity (BA) analyses indicated mountain goat males and females exhibit similar degrees of range fidelity during most of the year, though notable exceptions were evident. For example, females exhibited higher range fidelity during summer and winter, relative to males. Because females typically incur increased energetic demands associated with lactation during summer and also greater predation risk during summer and winter, relative to males, higher range fidelity is likely linked to benefits associated with increased knowledge about distribution of nutritional resources and predation‐risk refugia, similar to findings documented in other northern ungulates (Forrester et al., 2015; Peignier et al., 2019; Wittmer et al., 2006).

Seasonal variation in environmental conditions and reproductive requirements also exert a strong influence on range fidelity behavior. Similar to previous studies on caribou (Peignier et al., 2019) and deer (Hellickson et al., 2008; Igota et al., 2004), we documented lower range fidelity in winter relative to summer in mountain goats. This finding lends support to the hypotheses that variation in environmental conditions influences range fidelity behavior by altering predictability of key resources (Peignier et al., 2019). In high‐mid latitude environments, this dynamic may be most pronounced when winter snow buries food resources and alters the distribution, availability, and, ultimately, predictability of food resource distribution for herbivores (Fox, 1983; Gilbert et al., 2017; Peignier et al., 2019; White et al., 2009), a relationship that can be further complicated by the highly variable intra‐ and interannual frequency of winter snow events (Littell et al., 2018) that influence locomotory capabilities (Dailey & Hobbs, 1989) and movement dynamics (Richard et al., 2014b). Within this context, social and behavioral dynamics may further influence winter range fidelity via seasonal carry‐over effects. For example, male mountain goats exhibited the lowest degree of range fidelity in winter, a finding that may have been related to wide ranging and sometimes exploratory movements that occurred during the preceding rut period. Such movements may result in males ending up in unpredictable locations at the end of the rut, a moment that coincides with the onset of winter, and results in increased variability in range fidelity; especially if deep, locomotion‐inhibiting snow events occur in early winter and limit their ability to return to previously used seasonal core use wintering areas.

### 5.3 Effects of age on range fidelity

Older animals are expected to exhibit higher range fidelity due to increased experience and local knowledge of their surroundings, especially if past experience resulted in beneficial fitness‐linked outcomes such as increased reproductive success (Cameron & Linklater, 2007; Greenwood & Harvey, 1982; Testa et al., 2000). In our study, we documented limited support for age‐related effects on range fidelity in mountain goats, similar to previous investigations of black‐tailed deer (Forrester et al., 2015). For example, we anticipated that during the rut, prime‐aged and older males would exhibit increased range fidelity, relative to subadults, due to social dominance and previous mating experience. Instead, our findings revealed that range fidelity during the rut was relatively low, as compared to summer, and may reflect trade‐offs associated with optimizing success of mating opportunities in this polygynous species. Specifically, in some instances males, provided they are socially dominant, may be able to maximize breeding opportunities by concentrating use in areas of high female density; however, in most cases wide‐ranging exploratory movements are likely required to successfully procure mating opportunities with heterogeneously distributed receptive females across geographically complex landscapes. Consequently, previous experience and range fidelity may not foster substantial fitness benefits under such conditions, in contrast to cases where the distribution of prospective mates is highly predictable in space and time (i.e., Cameron & Linklater, 2007). In other seasonal contexts, age‐specific patterns in range fidelity were likewise weak and suggest that previous experience may not exert strong fitness benefits, perhaps because knowledge regarding the distribution of key resources may be acquired relative quickly, a plausible hypothesis given the relatively small home ranges used.

### 5.4 Modeling considerations, constraints, and inference

Familiarity with a local environment can represent an important element of habitat selection at broad spatial scales, and when coupled with positive reinforcement associated with selection for beneficial habitat attributes, a finer scale provides a mechanistic underpinning for range fidelity behavior (Wolf et al., 2009). Yet, disentangling resource selection behavior from range fidelity can be complex and incorporating explicit knowledge about resource selection and distribution of available resources, or habitat patches, can facilitate an enhanced understanding of range fidelity behavior (Lafontaine et al., 2017; van Moorter et al., 2009, 2013). For example, if habitat is extremely limited in availability, reuse of a given site may be mandatory and independent of previous experience or familiarity. To address these considerations, we used a study area‐specific resource selection function model to illustrate how habitat availability was not constrained, based on the movement capabilities and space‐use requirements of mountain goats (White, 2006; White et al. 2012; White and Gregovich, 2017; Figure S1). Specifically, we determined that mountain goats annually reused winter ranges 97% of the time, even though home ranges constituted as little as 4% of the available habitat. Thus, the chance of an animal randomly reusing a home range based on factors unrelated to previous experience or familiarity appears exceedingly small. While these results provide convincing evidence of range fidelity in our study, implementation of statistical approaches involving direct integration of resource selection and range fidelity processes may represent a promising complementary pathway for advancing our knowledge of range fidelity in the future (Lafontaine et al., 2017). For example, the utilization distribution overlap method used in this study provided a quantitative and relatively precise means for assessing how distributional patterns within seasonally reused home ranges were emulated from one year to the next. While current formulations of resource selection modeling approaches do not explicitly quantify and integrate utilization distribution overlap information, they can expand our knowledge of range fidelity behavior by evaluating the relative strength of fidelity versus. habitat characteristics in the context of broader investigation of resource selection behavior and prediction of a species distribution.

### 5.5 Conservation implications

The combined effects of having relatively small home ranges, especially during the critical winter and parturition season, and high range fidelity have important conservation implications for habitat specialists such as mountain goats. While a high degree of fidelity to seasonal home ranges does not necessarily imply a lack of behavioral flexibility, it is important to recognize that mountain goats exhibit an explicit recognition of important features in their local landscapes. Disruption or physical alteration of seasonal home ranges is likely to compromise an individual animal's ability to optimally utilize key resources such as forage patches or predator‐escape refugia, especially if they are limited in supply. Our analyses of range fidelity indicate that mountain goats perceive their environment and make space‐use decisions at both broad (i.e., returning to the same seasonal home range) and fine scales (i.e., reusing home ranges in a similar degree of spatial intensity) of spatial resolution. For a species that endure relatively extreme climatic conditions and is highly specialized to avoid the risk of predation by using spatially limited, discrete terrain features, it is important to recognize that anthropogenic impacts such as habitat alteration may disrupt range fidelity behavior (Faille et al., 2010; Richard & Côté, 2016) and capabilities, resulting in deleterious effects at the individual‐ and population‐level scales.

## CONFLICT OF INTEREST

None declared.

## AUTHOR CONTRIBUTIONS

**Yasaman N. Shakeri:** Conceptualization (lead); data curation (equal); formal analysis (lead); investigation (equal); methodology (supporting); resources (lead); visualization (lead); writing‐original draft (lead); writing‐review & editing (equal). **Kevin S. White:** Conceptualization (supporting); data curation (equal); formal analysis (supporting); funding acquisition (lead); methodology (equal); project administration (lead); visualization (supporting); writing‐original draft (supporting); writing‐review & editing (supporting). **Jason N. Waite:** Formal analysis (supporting); software (supporting); validation (lead).

## Supporting information

Supplementary Material

## Data Availability

Data are available from the Dryad Digital Repository: https://doi.org/10.5061/dryad.wdbrv15pg. The spatial data that support the findings of this study may be available on request from the corresponding author. The data are not publicly available due to privacy or ethical restrictions [Alaska Statue 16.05.815(d)].
