# Supplementary material for: Staying close to home: Ecological constraints on space use and range fidelity in a mountain ungulate
Source: Ecol Evol. 2021 Jul 9;11(16):11051–64. doi: 10.1002/ece3.7893 (PMC8366887; doi:10.1002/ece3.7893)

Supplemetal Materials:

Table S1. Summary of pairwise contrasts of female vs. male mountain goat home range sizes by age class and season. * indicates a significant difference at α= 0.05.

| Age Class | Season | Ratio | SE | p |  |
| --- | --- | --- | --- | --- | --- |
| Subadult | Kidding | 0.83 | 0.45 | 0.728 |  |
|  | Summer | 0.71 | 0.27 | 0.362 |  |
|  | Rut | 0.20 | 0.07 | < 0.001 | * |
|  | Winter | 0.80 | 0.26 | 0.487 |  |
|  |  |  |  |  |  |
| Adult | Kidding | 0.44 | 0.07 | < 0.001 | * |
|  | Summer | 1.49 | 0.22 | 0.008 | * |
|  | Rut | 0.27 | 0.04 | < 0.001 | * |
|  | Winter | 1.37 | 0.20 | 0.032 | * |
|  |  |  |  |  |  |
| Senior | Kidding | 0.75 | 0.28 | 0.445 |  |
|  | Summer | 1.48 | 0.50 | 0.249 |  |
|  | Rut | 0.33 | 0.12 | 0.002 | * |
|  | Winter | 0.97 | 0.33 | 0.936 |  |

Table S2. Summary of age class pairwise contrasts of mountain goats home range sizes by sex and season. * indicates a significant difference at α= 0.05.

| Sex | Season | Group 1 | Group 2 | Ratio | SE | p |  |
| --- | --- | --- | --- | --- | --- | --- | --- |
| Female | Kidding | Adult | Senior | 0.85 | 0.24 | 0.828 |  |
|  |  | Adult | Subadult | 0.72 | 0.27 | 0.645 |  |
|  |  | Senior | Subadult | 0.85 | 0.37 | 0.926 |  |
|  |  |  |  |  |  |  |  |
|  | Summer | Adult | Senior | 0.87 | 0.23 | 0.856 |  |
|  |  | Adult | Subadult | 0.96 | 0.28 | 0.991 |  |
|  |  | Senior | Subadult | 1.11 | 0.42 | 0.957 |  |
|  |  |  |  |  |  |  |  |
|  | Rut | Adult | Senior | 0.58 | 0.16 | 0.127 |  |
|  |  | Adult | Subadult | 0.97 | 0.25 | 0.993 |  |
|  |  | Senior | Subadult | 1.67 | 0.59 | 0.310 |  |
|  |  |  |  |  |  |  |  |
|  | Winter | Adult | Senior | 0.88 | 0.23 | 0.870 |  |
|  |  | Adult | Subadult | 0.98 | 0.24 | 0.997 |  |
|  |  | Senior | Subadult | 1.12 | 0.38 | 0.939 |  |
|  |  |  |  |  |  |  |  |
| Male | Kidding | Adult | Senior | 1.44 | 0.41 | 0.405 |  |
|  |  | Adult | Subadult | 1.35 | 0.57 | 0.756 |  |
|  |  | Senior | Subadult | 0.94 | 0.46 | 0.990 |  |
|  |  |  |  |  |  |  |  |
|  | Summer | Adult | Senior | 0.86 | 0.21 | 0.808 |  |
|  |  | Adult | Subadult | 0.46 | 0.12 | 0.008 | * |
|  |  | Senior | Subadult | 0.53 | 0.18 | 0.145 |  |
|  |  |  |  |  |  |  |  |
|  | Rut | Adult | Senior | 0.71 | 0.17 | 0.345 |  |
|  |  | Adult | Subadult | 0.71 | 0.18 | 0.354 |  |
|  |  | Senior | Subadult | 1.00 | 0.33 | 1.000 |  |
|  |  |  |  |  |  |  |  |
|  | Winter | Adult | Senior | 0.62 | 0.15 | 0.131 |  |
|  |  | Adult | Subadult | 0.57 | 0.13 | 0.044 | * |
|  |  | Senior | Subadult | 0.92 | 0.30 | 0.965 |  |

Table S3. Summary of seasonal pairwise contrasts of mountain goats home range sizes by sex and age class. * indicates a significant difference at α= 0.05.

| Sex | Age Class | Group 1 | Group 2 | Ratio | SE | p |  |
| --- | --- | --- | --- | --- | --- | --- | --- |
| Female | Subadult | Kidding | Rut | 1.63 | 0.62 | 0.581 |  |
|  |  | Kidding | Summer | 0.75 | 0.30 | 0.892 |  |
|  |  | Kidding | Winter | 2.45 | 0.93 | 0.089 |  |
|  |  | Rut | Summer | 0.46 | 0.15 | 0.076 |  |
|  |  | Rut | Winter | 1.50 | 0.44 | 0.494 |  |
|  |  | Summer | Winter | 3.26 | 1.05 | 0.001 |  |
|  | Adult | Kidding | Rut | 1.21 | 0.17 | 0.555 |  |
|  |  | Kidding | Summer | 0.56 | 0.08 | < 0.001 | * |
|  |  | Kidding | Winter | 1.80 | 0.25 | < 0.001 | * |
|  |  | Rut | Summer | 0.47 | 0.06 | < 0.001 | * |
|  |  | Rut | Winter | 1.49 | 0.20 | 0.013 |  |
|  |  | Summer | Winter | 3.20 | 0.42 | < 0.001 | * |
|  | Senior | Kidding | Rut | 0.83 | 0.28 | 0.941 |  |
|  |  | Kidding | Summer | 0.57 | 0.19 | 0.326 |  |
|  |  | Kidding | Winter | 1.85 | 0.60 | 0.225 |  |
|  |  | Rut | Summer | 0.69 | 0.22 | 0.668 |  |
|  |  | Rut | Winter | 2.24 | 0.69 | 0.044 | * |
|  |  | Summer | Winter | 3.23 | 1.01 | 0.001 | * |
|  |  |  |  |  |  |  |  |
| Male | Subadult | Kidding | Rut | 0.39 | 0.17 | 0.148 |  |
|  |  | Kidding | Summer | 0.64 | 0.29 | 0.766 |  |
|  |  | Kidding | Winter | 2.37 | 1.04 | 0.206 |  |
|  |  | Rut | Summer | 1.65 | 0.52 | 0.372 |  |
|  |  | Rut | Winter | 6.08 | 1.76 | < 0.001 | * |
|  |  | Summer | Winter | 3.68 | 1.10 | < 0.001 | * |
|  | Adult | Kidding | Rut | 0.74 | 0.09 | 0.049 | * |
|  |  | Kidding | Summer | 1.89 | 0.22 | < 0.001 | * |
|  |  | Kidding | Winter | 5.58 | 0.66 | < 0.001 | * |
|  |  | Rut | Summer | 2.56 | 0.28 | < 0.001 | * |
|  |  | Rut | Winter | 7.57 | 0.82 | < 0.001 | * |
|  |  | Summer | Winter | 2.95 | 0.32 | < 0.001 | * |
|  | Senior | Kidding | Rut | 0.37 | 0.12 | 0.012 | * |
|  |  | Kidding | Summer | 1.13 | 0.36 | 0.981 |  |
|  |  | Kidding | Winter | 2.40 | 0.80 | 0.043 | * |
|  |  | Rut | Summer | 3.10 | 0.92 | 0.001 | * |
|  |  | Rut | Winter | 6.59 | 1.96 | < 0.001 | * |
|  |  | Summer | Winter | 2.12 | 0.64 | 0.059 |  |

**
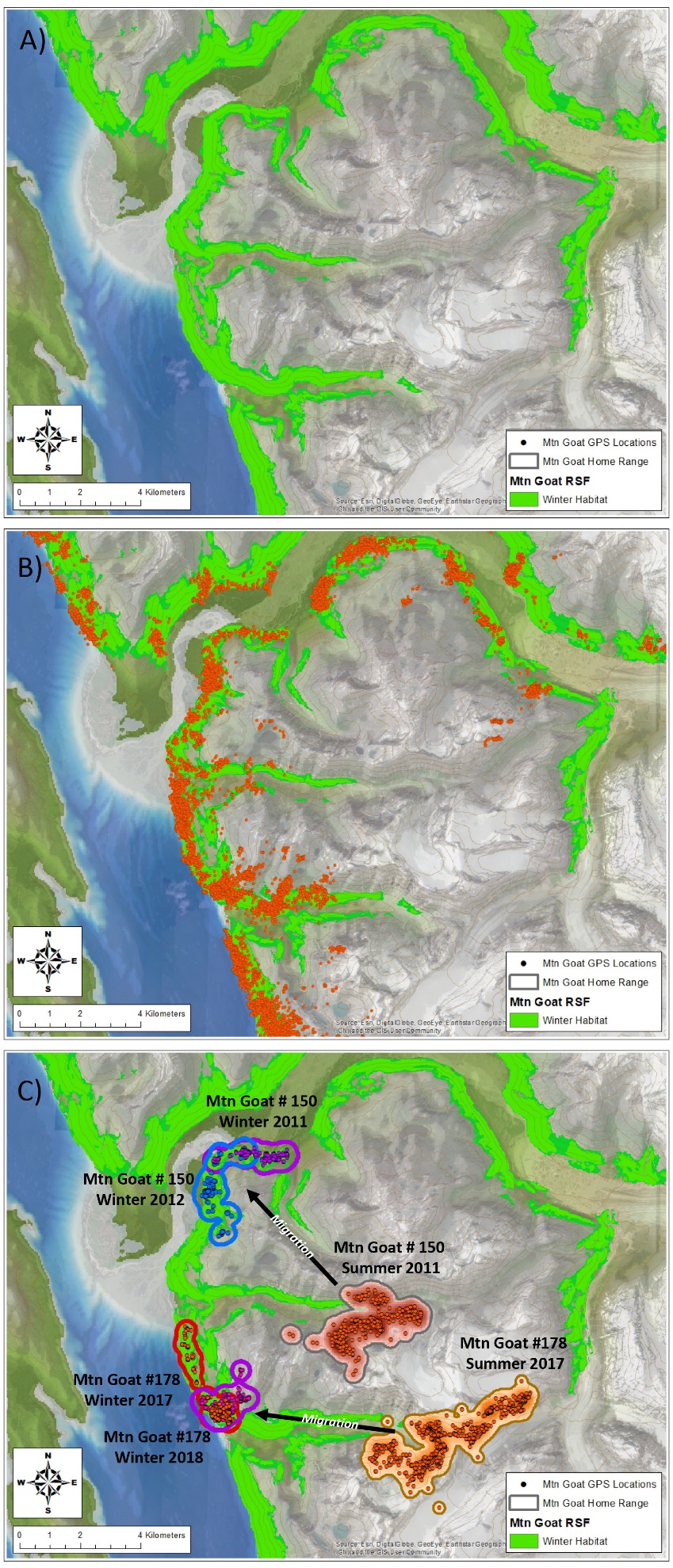
Figure S1.** Maps describing the distribution of mountain goat winter habitat and use by GPS radio-collared individuals. A) Map depicting the distribution of mountain goat winter habitat (light green shaded area) in the southern part of the study area, near the Katzehin River, AK. Delineated habitat is based on RSF analyses with habitat models predicting winter habitat at a high degree of validation accuracy, as determined via k-fold cross-validation (White et al 2012). B) Map juxtaposing winter GPS locations (red dots) from all mountain goats monitored (n = 48 individuals, 28,128 locations, 2005-2020) in the area illustrating widespread use of delineated winter habitat (incomplete use of habitat likely due marking only 5-10% of animals in area). C) Map depicting seasonal space use of two example mtn goats (#150 and #178). Seasonal home ranges and associated GPS radio-collar locations are color coded and annotated. Winter home range area is transparent to illustrate underlying distribution of winter habitat. The examples illustrate the specific winter ranges used and how they are re-used the following year, in the context of the available winter habitat. The individual mountain goats had access to suitable winter habitat in many different areas but utilized home ranges that were previously used and that tightly conformed to delineated winter range. This is a conservative representation since mountain goats have the capability to move much further between summer and winter range than illustrated in the examples.

Figure S2. Mountain goat home range (95% fixed kernel) estimates in relation to sex, age category and season during 2005 – 2016 in upper Lynn Canal, AK.


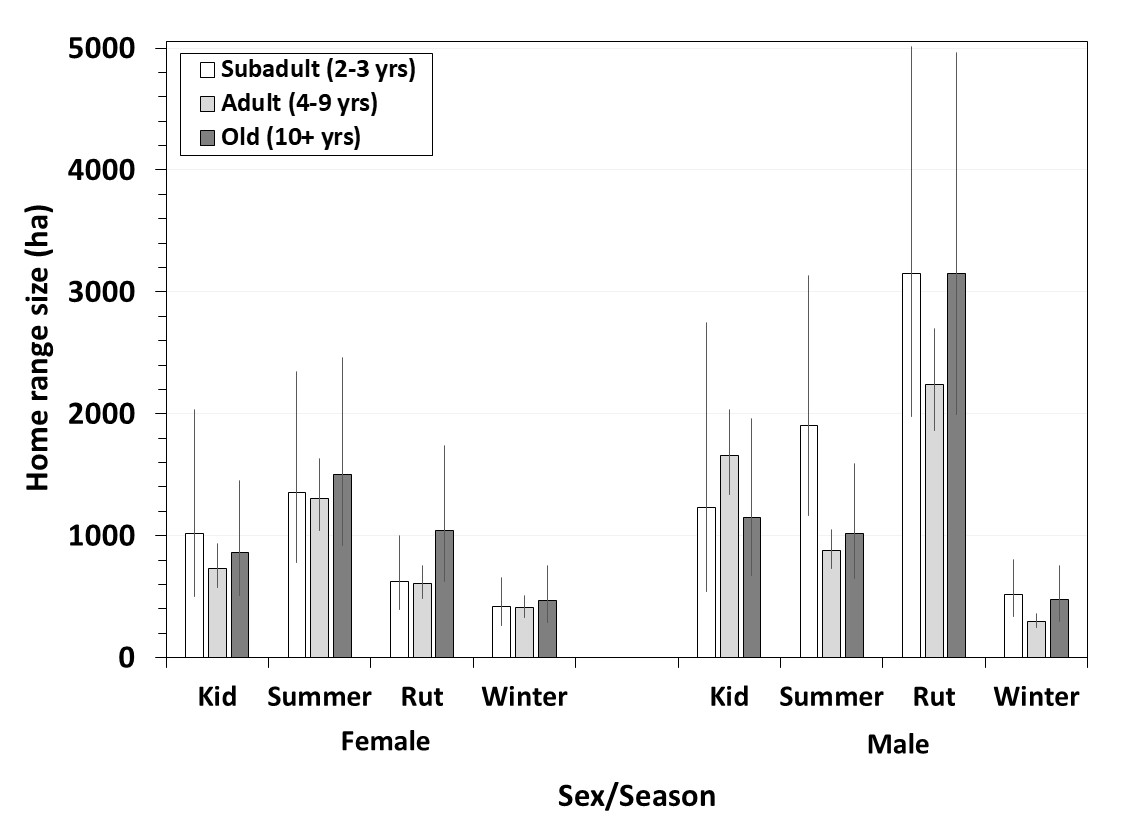


Figure S3. Mountain goat home range utilization distribution overlap (BA) estimates in relation to sex and season during 2005 – 2016 in upper Lynn Canal, Alaska. Utilization distribution overlap characterizes the similarity of seasonal home range utilization distributions for given individual during consecutive years; calculated using Bhattacharyya’s affinity (BA) estimator.


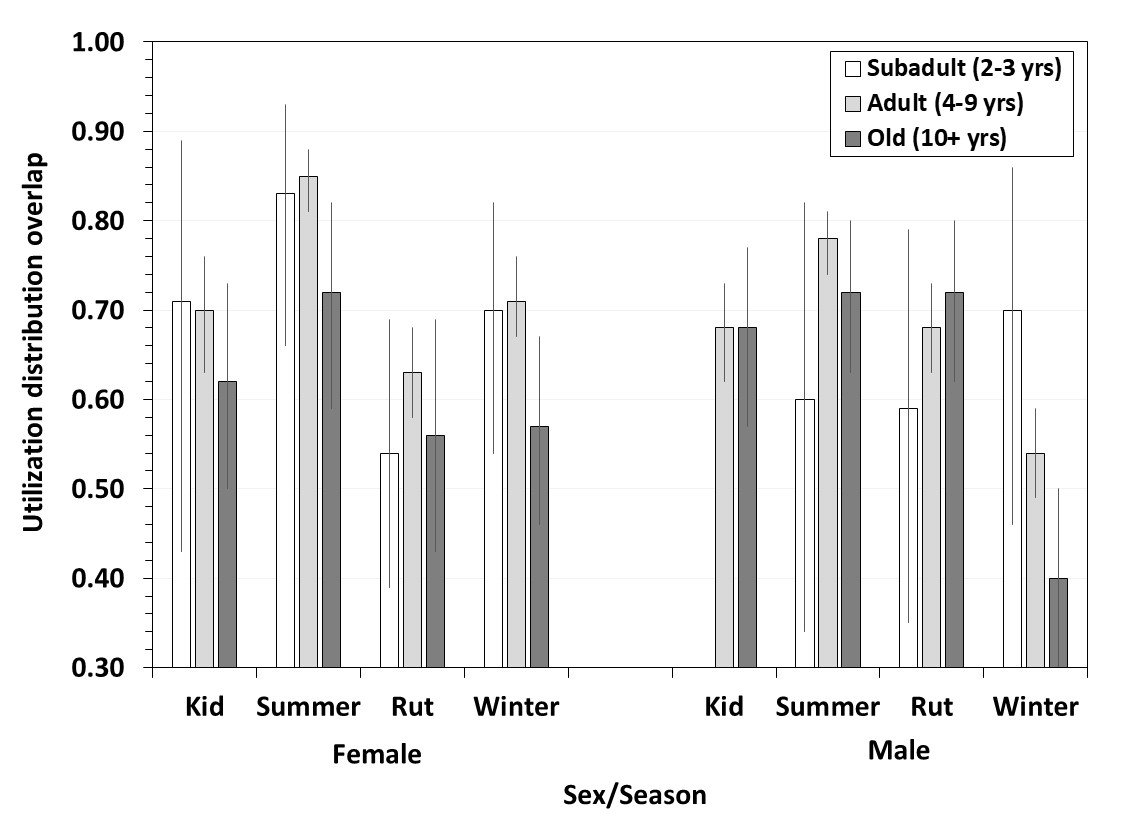

Supplement: Supplementary file 1 — Supplementary Material [file ECE3-11-11051-s001.docx]
